# Efficacy and cost-effectiveness of outdoor residual spraying with α-cypermethrin, pirimiphos-methyl, and deltamethrin against sand flies: a pragmatic cluster randomised controlled field trial in Uganda

**DOI:** 10.1186/s41182-026-00985-9

**Published:** 2026-05-27

**Authors:** Kamoga Livingstone, Edwin Kigozi, Nelson Ssewante, Blaise Kiyimba, Shamim Nabidda, Elvis Seremba, Andrew Munerya, Richard Achuma, Damiano Lomokol, Nantume Olivia, Patrick Banadda Matovu, Daniel Tamale, Patrick Sagaki, Joshua Epuitai, Felix Bongomin, Scovia Nalugo Mbalinda

**Affiliations:** 1 Department of Nursing, College of Health Sciences, Makerere University, Kampala, Uganda; 2 Centre On Climate Change and Planetary Health, London School of Hygiene and Tropical Medicine, Keppel Street, London, WC1E 7HT UK; 3 Child Health and Development Centre, College of Health Sciences, Makerere University, Kampala, Uganda; 4 Department of Medicine, College of Health Sciences, Makerere University, Kampala, Uganda; 5 Department of Nursing and Midwifery, Aga Khan University, Kampala, Uganda; 6 Amudat Church of Uganda Hospital, Amudat District Local Government, Amudat, Uganda; 7 Department of Nursing, Busitema University, Mbale, Uganda; 8 Department of Medical Microbiology and Immunology, Faculty of Medicine, Gulu University, Gulu, Uganda; 9 Department of Pharmacy, Kampala School of Paramedicals, Kayunga, Uganda

**Keywords:** Visceral leishmaniasis, Insecticides, Kala Azar, Amudat, East Africa

## Abstract

**Introduction:**

Visceral leishmaniasis (VL) is a significant public health challenge in East Africa. Achieving the WHO 2030 target of reducing VL mortality to below 1% requires novel control strategies. In this study, we evaluated the efficacy and cost-effectiveness of three insecticide formulations for outdoor residual insecticide spraying (ORS) in selected villages of Amudat district, Uganda.

**Methods:**

A pragmatic clustered randomised controlled field trial was conducted in five clusters. Three clusters were randomly allocated to receive ORS with either α-cypermethrin, pirimiphos-methyl, or deltamethrin, while two clusters served as untreated controls. The primary outcome was the change in sandfly counts per trap-night, measured using CDC light and sticky traps before and after the intervention. These were compared using the Wilcoxon signed-rank test to assess reductions independent of other villages. Then, we used negative binomial regression with a log link of trap-nights to model sandfly counts, which were included as an offset to account for variation in sampling effort.

**Results:**

After spraying the insecticides in the intervention villages, the sandfly population declined by 62%, 60%, and 49% with α-cypermethrin, pirimiphos-methyl, and deltamethrin, respectively. Sandfly captures increased by fourfold (353%) in the control villages in the same period. Using a negative binomial regression with village-clustered standard errors, α-cypermethrin reduced sandfly counts by 92% compared to the change in control villages (IRR = 0.08, 95% CI 0.07–0.38, *p* < 0.001). Pirimiphos-methyl and deltamethrin reduced counts by 91% (IRR = 0.09, 95% CI 0.07–0.45) and 89% (IRR = 0.11, 95% CI 0.10–0.56), respectively. A cost-effectiveness analysis showed that α-cypermethrin emerged as a more cost-effective option, followed by deltamethrin and lastly pirimiphos-methyl, with annual cost per village of $382, $390 and $408, respectively.

**Conclusion:**

Our findings provide tentative observations that may guide vector control programmes. The relatively high efficacy and favourable cost-effectiveness of α-cypermethrin are suggestive that it could be a suitable option for inclusion in integrated vector management strategies targeting outdoor sandfly populations in this region.

**Supplementary Information:**

The online version contains supplementary material available at 10.1186/s41182-026-00985-9.

## Introduction

Visceral leishmaniasis (VL), also known as Kala Azar but locally referred to as Termes in the Pokot language, is a serious and life-threatening neglected tropical disease and a significant public health challenge in East Africa. The region is a global hotspot, accounting for an estimated 66% of the global VL burden [1]. In Uganda, the disease persists with a concerning case fatality rate of 5%, disproportionately affecting children under 14 years of age [2]. The heaviest burden falls on endemic communities already grappling with poverty, malnutrition, and fragile health systems, underscoring the need for effective and sustainable preventive strategies [3, 4].

Vector control through the reduction of sandfly populations is a cornerstone of primary VL prevention. While insecticide-treated bed nets have demonstrated efficacy against the primary vector, *Phlebotomus orientalis/martini*, evidence increasingly shows that this single intervention is insufficient for optimal disease control [4, 5]. This is particularly true in pastoralist communities in northeastern Uganda, such as the Pokot and Karimajong, whose livelihoods shape unique risk behaviours. Practices such as sleeping outdoors during hot seasons and resting on or near anthills and *Acacia seyal* and *Balanites aegyptiaca* trees, known sandfly habitats, while grazing cattle, significantly increase exposure to daytime sandfly bites, thereby limiting the protective effect of bed nets [6, 7].

Consequently, achieving the WHO’s 2030 target of reducing VL mortality to below 1% necessitates the integration of complementary vector control strategies that target outdoor transmission [7]. Outdoor residual spraying (ORS) represents a promising approach, with demonstrated potential to suppress sandfly populations in peridomestic and outdoor environments [8]. However, the deployment of ORS in Uganda has been minimal, and a critical evidence gap remains. There is a stark lack of robust, local data on the comparative efficacy and cost-effectiveness of different insecticide classes for ORS to inform evidence-based policy and implementation.

This gap is especially pressing in northeastern Uganda, a region ecologically confirmed as a major VL transmission focus [9]. To address this, we conducted a pragmatic cluster randomised field trial to evaluate the efficacy and cost-effectiveness of three insecticide formulations, α-cypermethrin, pirimiphos-methyl, and deltamethrin for ORS against sandfly populations in the endemic communities of Amudat District, northeastern Uganda.

## Material and methods

### Study design

This was a pragmatic cluster randomised controlled field trial (CRCT) study with five arms: three interventional and two control cluster arms, conducted over 60 days. The first 30 days between 7th May 2025 to 7th June 2025 constituted the pre-intervention phase, dedicated to baseline sandfly trapping. The spraying was then applied on the same day in all intervention clusters. The final 30 days between 8th June 2025 to 8th July 2025 constituted the post-intervention phase for follow-up trapping. Clusters were villages located in the Amudat District, northeastern Uganda, each having at least 15 households.

### Study area

**Fig. 1:**
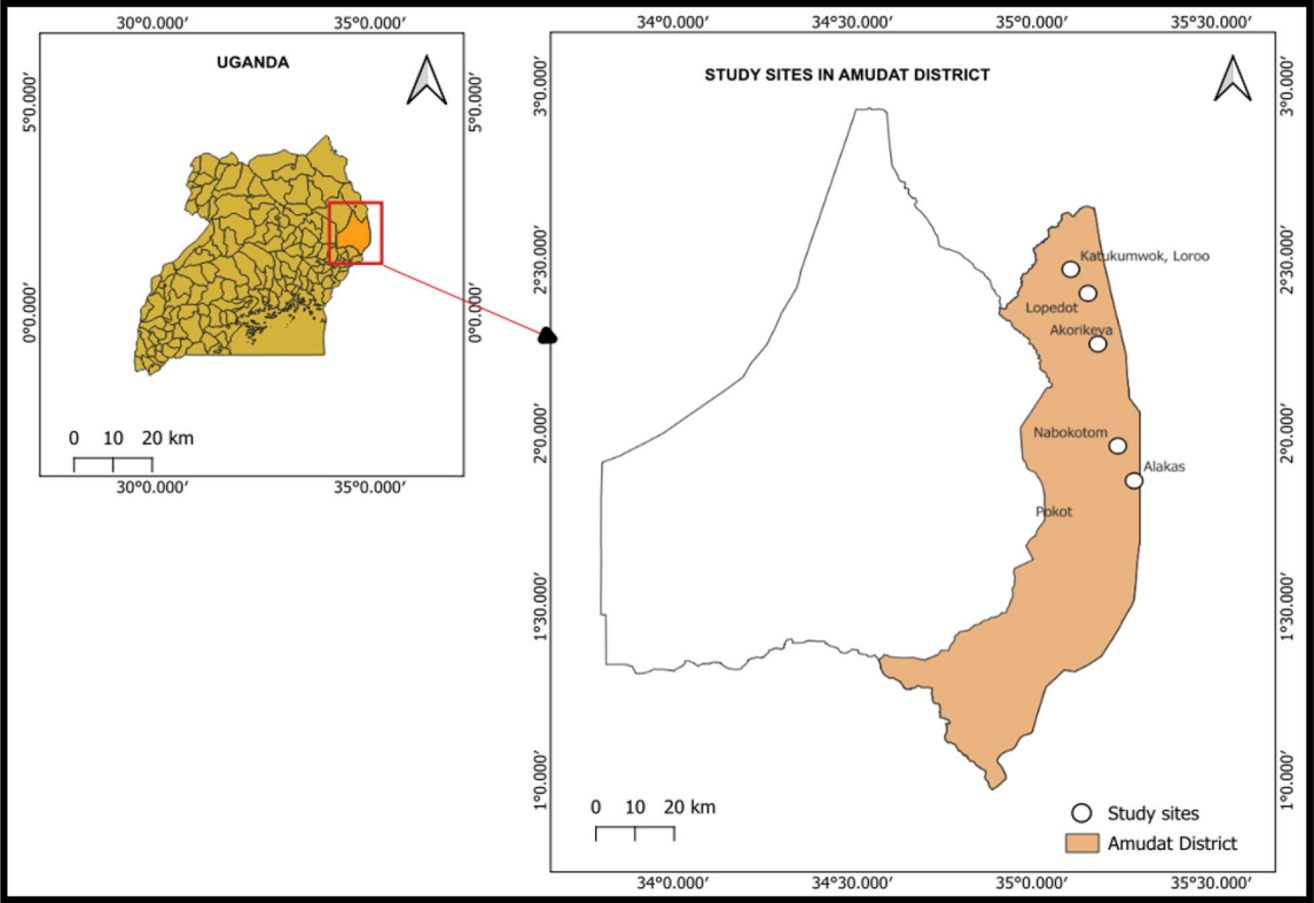
A map of Amudat District showing the different study sites

This study was carried out in the Amudat District, located in northeastern Uganda (Fig. 1). It is located approximately 400 kms by road northeast of Kampala, Uganda’s capital. The district has an estimated population of 203,358 people, predominantly the Pokot [10]. The Pokot people are known for their pastoralist lifestyle, with livestock, particularly cattle, goats, and camels, forming the backbone of their economy. The district is one of the endemic foci for visceral leishmaniasis in the region and harbours the highest number of sand flies in the country [9, 11]. The district is served by the Amudat Church of Uganda Hospital, which receives all referral cases from lower-level health facilities in the district.

### Selection of the villages

A formal sample size calculation for the primary outcome (sandfly count) was not feasible due to the lack of prior data on the variance of sandfly counts in this setting. The number of clusters was therefore determined pragmatically, based on logistical constraints and following the design of similar CRCTs in vector control [12]. We aimed to include a minimum of 4–6 clusters to ensure a reasonable balance between intervention and control arms.

Prior to the commencement of field activities, hospital records for the Amudat Church of Uganda Hospital were reviewed to identify the number and source of VL cases recorded in the last two years. A total of 59 leishmaniasis cases were identified from the hospital records in the selected villages for the period of January 2023 to December 2024. A village was eligible for selection if it had at least one case of VL reported at this hospital in the past two years. A list of all eligible villages was developed, from which a total of five villages were randomly selected.

### Cluster characteristics

All study clusters were inhabited by the Pokot, a pastoralist community that rears livestock such as cattle, goats, sheep, and camels. These animals are typically grazed in open areas and kept in kraals within household compounds, often surrounding the living huts; in some cases, smaller animals may also be kept inside the huts. The homesteads are commonly surrounded by acacia trees and other shrubs.

### Randomisation

Before initiation of the sandfly capture activities, simple randomisation was conducted to assign the selected villages (clusters) to either the intervention (three clusters) or control (two clusters) arms. An independent individual who was not involved in any aspect of the study generated a list of random allocation codes using a simple random number generator. Each cluster was assigned a unique code, and the codes were then randomly drawn and matched to the intervention or control slots. The final allocation for each cluster was placed in sequentially numbered, opaque, sealed envelopes (SNOSE) and kept by this independent individual.

**Fig. 2:**
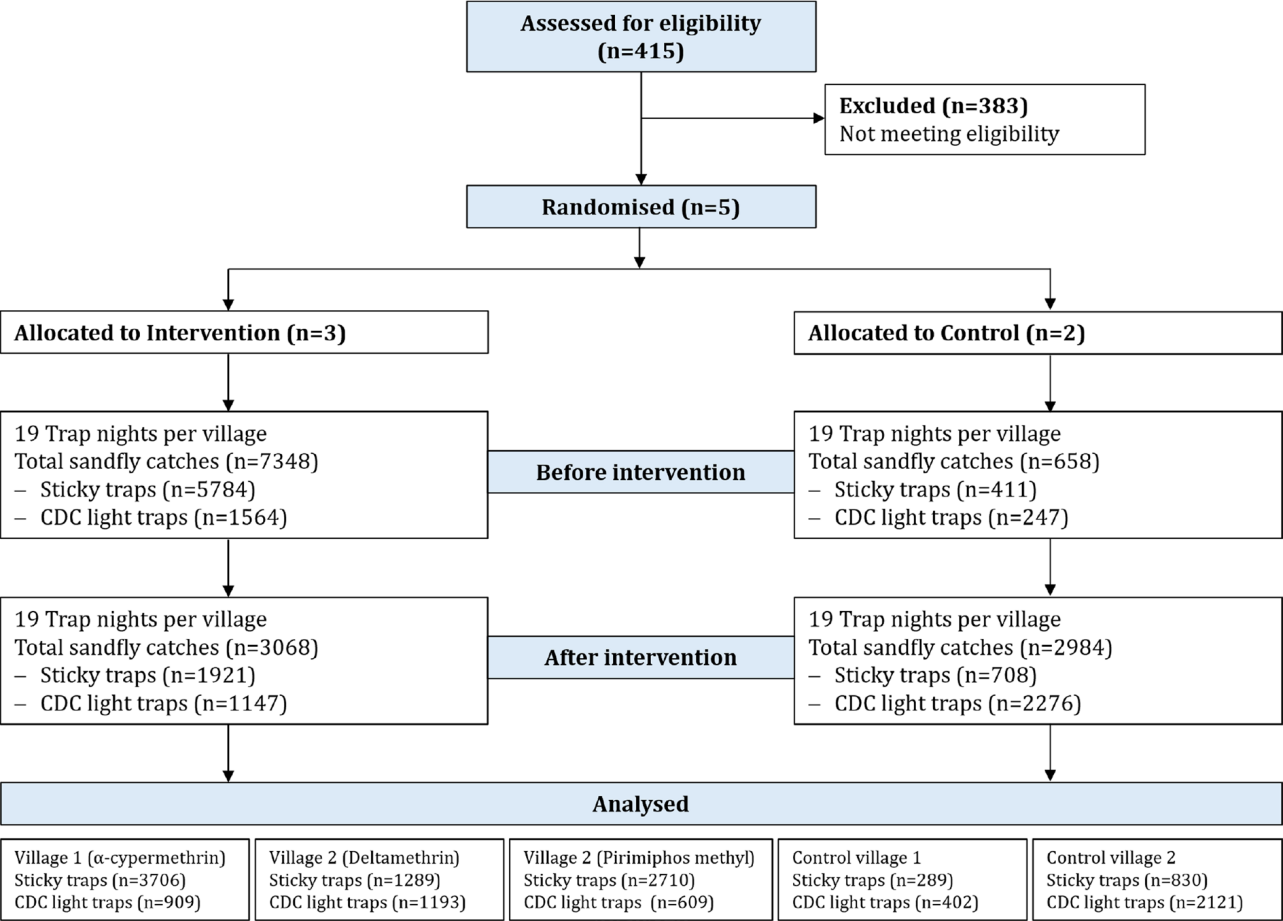
Study consort diagram

The study team, including investigators, remained fully blinded to the allocation until completion of all pre-intervention procedures, specifically the first 30 days of baseline sandfly trapping. Once these activities were concluded, the envelopes were opened to reveal the assignments: Lopedot, Katukumwok, and Akorikeya were allocated to the three intervention arms, while Alakas and Nabokotom were allocated to the control arm (Fig. 2). However, the sprayers were aware of the insecticide being sprayed, and the community members knew when their communities were being sprayed. This could have introduced performance and reporting biases. Therefore, our results should be interpreted with that in mind. All activities were based on the Spirit and Consort guidelines for protocol development and reporting [13, 14].

### Insecticide application

The first cluster received 10% EC α-cypermethrin (100g/litre active ingredient (a.i) manufactured by HIL (India) Limited) at a dose of 25mg a.i/m^2^. The second cluster received pirimiphos-methyl (300mg/litre active ingredient capsule suspension manufactured by Syngenta, Env Global-Switzerland) at a dose of 1g a.i/m^2^, while the third cluster received deltamethrin (25g/litre active ingredient manufactured by Hemani Industries Limited-India) at a dose of 12.5mg a.i/m^2^. These insecticides were applied on the outside of the living houses (for both humans and animals), the surrounding compounds, perimeter fences of the kraals, in the courtyards, near the animal excreta dumpsites and under the *Balanites aegyptiaca* and *Acacia seyal* trees in the communities using IK spray pumps (Model IK INOX 10, 10 L capacity). A trained entomologist mixed the insecticides according to established guidelines, and trained sprayers wore personal protective equipment (PPE) during the application process. Nothing was sprayed in the control villages. After the application of the insecticides, clusters were monitored for any adverse environmental or household safety events such as respiratory irritation, skin reactions and effects on domestic animals. No adverse events were observed during the 24–96 h post-application period, based on household interviews and field inspections. The sprayed areas were measured using a tape measure to determine the surface areas where the chemicals were applied. While the water source itself was free, costs associated with its procurement and transportation from wells and boreholes were factored into the labour and transport expenditures. The other costs were determined from the transportation to the fields, and the costs for buying the chemicals on the market.

### Study procedures

In both the intervention and pre-intervention phase each of the five clusters received a single CDC light trap (model 512 by John W. Hock Company) which was set 0.5 m above the ground supported by a pole on the perimeter fences next to animal kraals, in the compound, also supported on the branch of the *Balanites aegyptiaca* and *Acacia seyal* trees as the *phlebotomine* sand flies are known to live in these trees and in the moist environment created by the animal excreta [12, 15]. A set of 15–20 sticky fly traps made from A4-size white papers coated with sunflower oil was deployed 0.5–1 m apart in each cluster where they were set horizontally on the cracked black cotton soils, near termite mounds, perimeter fences of the kraals and dumping sites of the animal excreta (dung), which are also breeding grounds for the phlebotomine flies [12, 16]. It is known that the effectiveness of the sticky fly traps is affected by their positions, so these were set horizontally rather than vertically because the horizontal position is more effective than the vertical [8] in the various communities to trap the sand flies. Both traps were set from 6 pm to 6 am every day and were rotated in all the known sandfly habitat areas in the clusters until the study period was over.

The villages in the control groups were not sprayed with any of the insecticides, and all the village members were sensitised by the research team with the help of the community health care workers not to apply any insecticide, which could interfere with the study’s results. The study worked with an experienced entomologist during the execution of the study protocol.

### Sorting of the sandflies

The captured sandflies were sorted every morning from the sticky fly trap using a small, fine brush, washed with detergent, and preserved in 70% ethanol. Those from the CDC light traps were knocked out using nitric acid and preserved. These were processed for sex and genus under a dissecting microscope, and the *Sergentomyia sp*.* w*ere counted without further processing and were not included in the results. The phlebotomine sandflies were morphologically identified to species level using established taxonomic keys[17, 18], with a focus on known VL vectors such as *Phlebotomus martini* and *P. orientalis*.

### Quality control

The collected data were stored on the principal investigator’s personal computer, secured with password encryption, and accessible only to the study team. The research team received training from senior colleagues to ensure the collection of high-quality data. Sprayers were trained by a qualified entomologist, who was overseeing their activities under the supervision of the investigators. All hard copies of data were securely stored in a locked cabinet, accessible only to the study team.

### Data analysis

Upon completion of data collection, data were downloaded from Kobo Collect and imported into Microsoft Excel for cleaning. Sandfly counts by sticky and CDC light traps were summed up to generate total counts before and after spraying for each study site. The cleaned dataset was then imported into STATA 14.2 for analysis. However, the graphs were constructed using GraphPad Prism 10.4.2.

Within each village, sandfly counts at a trap-night level before and after spraying were compared using the Wilcoxon signed-rank test to assess reductions independent of other villages.

Then, we used negative binomial regression (since counts were over-dispersed) with a log link of trap-nights to model sandfly counts, which were included as an offset to account for variation in sampling effort. The key explanatory variables were time (before- vs. after-spraying), chemical group (α-cypermethrin, pirimiphos-methyl, deltamethrin, or control), and their interaction term (time × chemical). This interaction estimates the difference-in-differences (DID) effect of each chemical relative to the control. Given the small number of clusters (*N* = 5), we employed a Wild Cluster Bootstrap with a 6-point Webb weight distribution to derive *p*-values and confidence intervals. This approach is specifically recommended for its superior performance and reliability when working with a limited number of clusters [19]. Model coefficients were exponentiated to produce incidence rate ratios (IRRs) with 95% confidence intervals, with IRRs < 1 indicating a reduction in sandfly counts. To account for temporal dependency in daily captures, we assessed model residuals using the autocorrelation function (ACF). No significant serial correlation was detected. Detailed model outputs are provided in Supplemental File 1. All statistical tests were two-tailed, and significance was set at *p* < 0.05.

## Cost-projection modelling

We applied an ingredient-based costing approach to estimate the resources required for a potential large-scale implementation of each insecticide intervention. To ensure generalisability, only costs directly related to an ideal implementation scenario were included, while research-specific expenditures were excluded. The cost components considered were: purchase of insecticides, field transport, labour, and spraying equipment.

To provide a standardised comparison, we projected costs based on a hypothetical annual cycle of two rounds of residual spraying, a frequency commonly recommended for sustained community-level sandfly control [20]. The estimated costs were adjusted for inflation using the Consumer Price Index (CPI) for April 2025, and these could have changed significantly over the last 11 months. Finally, all values were converted to United States Dollars (USD) using the exchange rate at the time of procurement (March–April 2025). We calculated the costs, that is: estimated cost per village: obtained by summing up all the costs incurred during application of the insecticide in the village.

Projected annual cost per village: above cost times two (based on recommended treatment cycles annually).

Projected annual cost per square metre: obtained by dividing the projected annual cost by the square metres for the respective village sprayed.

Projected annual cost per sandfly reduced: dividing the projected annual cost by the effective number of sandflies reduced by the insecticide. This was used to compare the performance of the three insecticides.

## Results

**Fig. 3:**
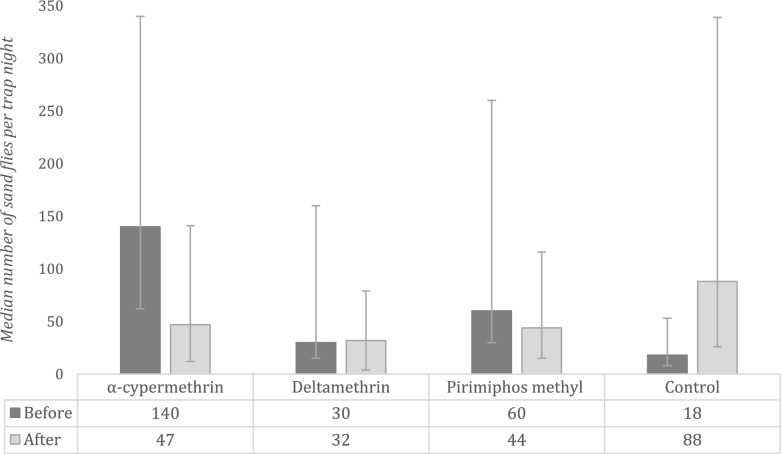
Sandfly counts per trap-night before and after spraying in each study village

Five villages (Fig. 2) were included in this study, three as intervention sites. Intervention villages included Lopedot, which received α-cypermethrin, while Akorikeya and Katukwomok received deltamethrin and pirimiphos methyl, respectively. Before spraying, Lopedot (α-cypermethrin) had a median of 140 (IQR: 78, 200) sand flies per trap-night, while the medians were 30 (15,130), 60 (30,200), 3(0,5) and 17 (10,30) in Akorikeya (deltamethrin), Katukwomok (pirimiphos-methyl) and both control villages combined, respectively (Table 1). After spraying, sandfly captures were reduced in the intervention villages, while they increased exponentially in the control villages (Fig. 3).

**Table 1:** Sandfly counts per study site aggregated by study phase

Site	Insecticide used	Number of Sandflies collected				
		Before spraying		After spraying		*p*-value^a^
		Total	Median (IQR)	Total	Median (IQR)	
Lopedot	α-cypermethrin	3341	140 (78,200)	1274	47 (35,94)	0.0126
Akorikeya	Deltamethrin	1643	30 (15,130)	839	32 (28,47)	0.6009
Katukwomok	Pirimiphos-methyl	2364	60 (30,200)	955	44 (29,72)	0.0733
Alakas	None	68	3 (0,5)	623	27 (9,42)	0.0003
Nabokotom	None	590	17 (10,30)	2361	70 (30, 228)	0.0017

^a^*P*-values derived from Wilcoxon signed-rank tests for within-village comparisons

## Efficacy of insecticides on sandfly counts

**Fig. 4:**
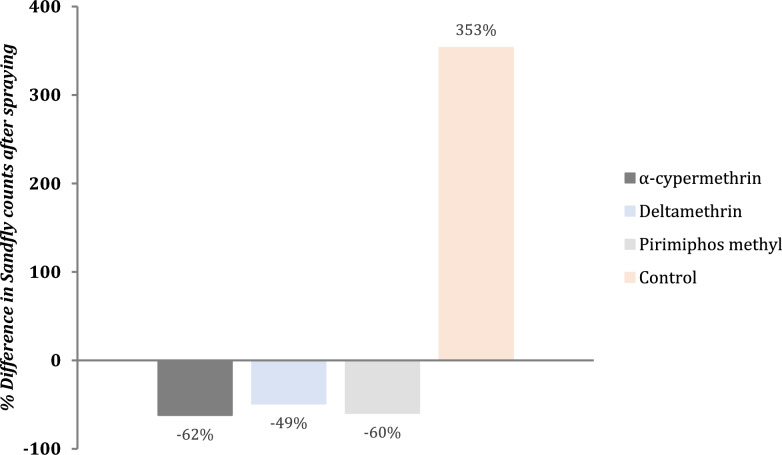
Percentage reduction in sandfly counts by insecticide type relative to the control

Upon application of the insecticides in the intervention villages, the total sandfly captures decreased by 62%, 60%, and 49% with α-cypermethrin (Lopedot village), pirimiphos-methyl (Katukwomok village), and deltamethrin (Akorikeya village), respectively. Conversely, sandfly captures increased by fourfold (353%) in the control villages during the same period (Fig. 4).

Based on the Wilcoxon signed-rank test, the reduction in the sandfly catches observed was statistically significant in Lopedot village, where α-cypermethrin was applied (*p* = 0.0126), but not in Akorikeya (deltamethrin) (*p* = 0.6009) and Katukwomok (pirimiphos-methyl) *p* = 0.0733) (Table 1).

Using a negative binomial regression with village-clustered standard errors, α-cypermethrin reduced sandfly counts by 92% compared to the change in control villages (IRR = 0.08, 95% CI 0.07–0.38, *p* < 0.001). Pirimiphos-methyl and deltamethrin reduced counts by 91% (IRR = 0.09, 95% CI 0.07–0.45) and 89% (IRR = 0.11, 95% CI 0.10–0.56), respectively (Table 2). While control villages exhibited a significant seasonal increase in sandfly density during the post-intervention period, this trend was effectively suppressed in the treatment villages (Fig. 6). Specifically, the predicted margins illustrate a sharp divergence in population trajectories: whereas the sandfly population in the control villages grew nearly fourfold, all insecticide-treated sites significantly reduced baseline levels.

**Table 2:** Comparison of the impact of insecticides on sandfly counts relative to changes in control villages

Variable	IRR	95% Bootstrap CI	*p*-value
Time (post-intervention vs. pre)	2.16	1.33–3.48	0.002
Main effects (insecticide relative to control)			
Control	Reference		
α-cypermethrin	10.16	3.61–12.95	< 0.001
Pirimiphos-methyl	7.19	2.39–8.89	< 0.001
Deltamethrin	4.99	1.66–6.18	0.001
Treatment effect (interaction)			
Control	Reference		
α-cypermethrin	0.08	0.07–0.38	< 0.001
Pirimiphos-methyl	0.09	0.07–0.45	< 0.001
Deltamethrin	0.11	0.10–0.56	0.001

*IRR* incidence rate ratio, *CI* confidence interval

## Cost of the insecticides used

**Fig. 5:**
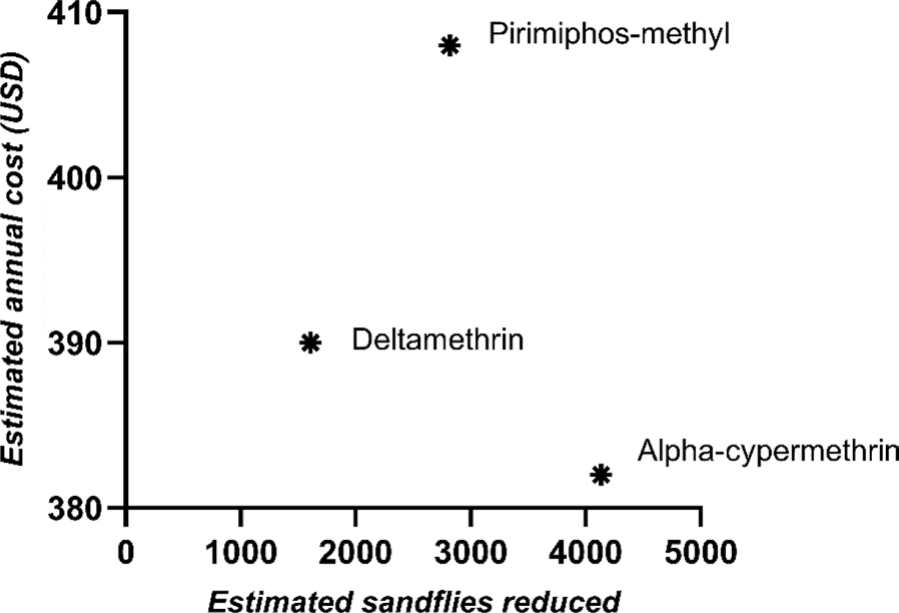
Scatter plot of the estimated annual cost relative to sandfly reduction estimates

**Fig. 6:**
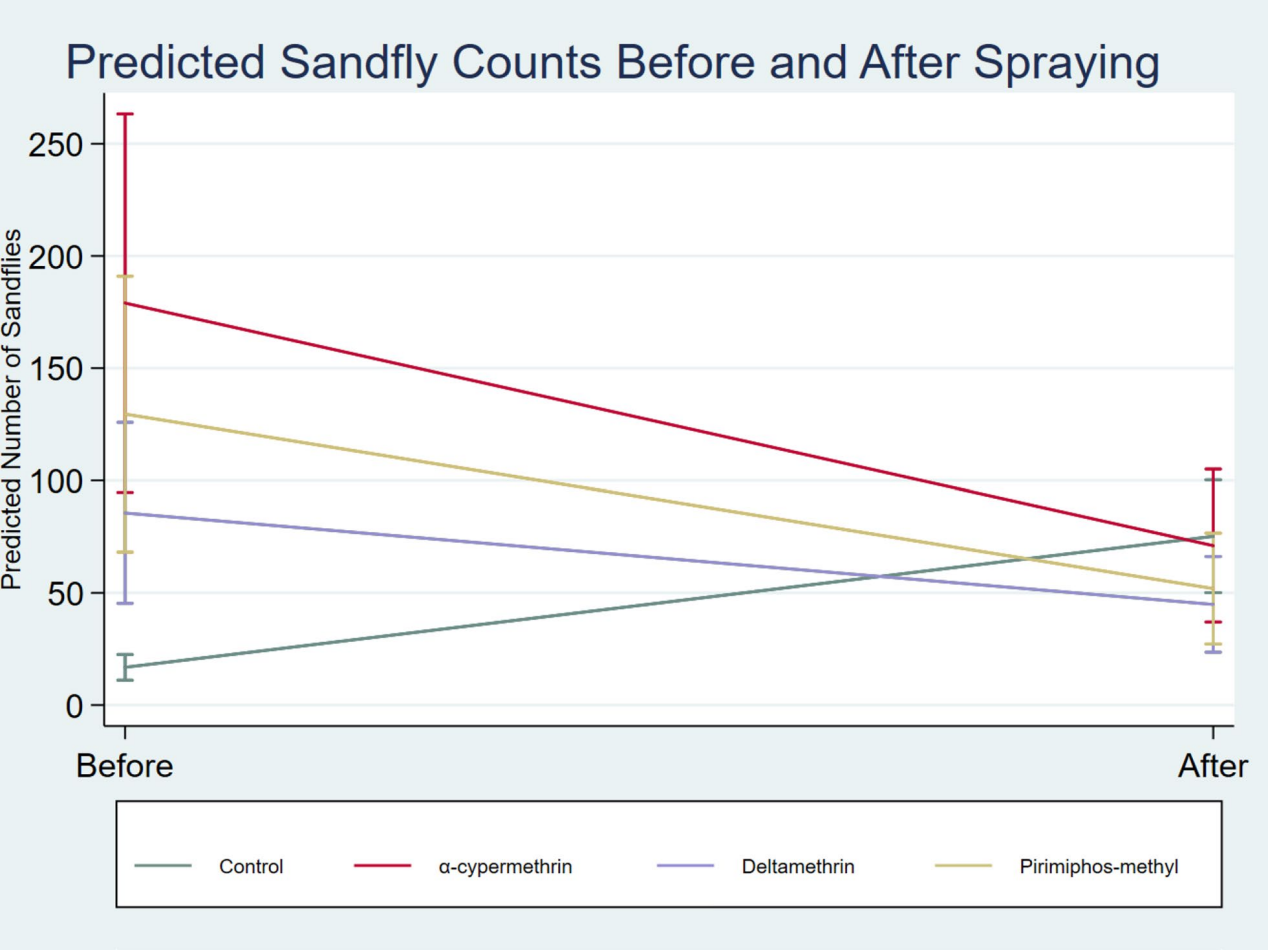
Predicted sandfly counts before and after spraying

Based on the cost projection modelling, α-cypermethrin was the cheapest to apply, followed by deltamethrin and lastly pirimiphos-methyl, with an estimated cost of $382, $390 and $408 per spray, respectively (Table 3). However, considering their respective effects on the sandfly populations in the respective villages, pirimiphos-methyl slightly had a better value for money compared to deltamethrin, valued at $0.58 per sandfly eliminated compared to deltamethrin’s $0.97. Overall, α-cypermethrin had the best value for money, killing more sandflies per unit dollar than the other insecticides (Figs. 5 and 6).

**Table 3:** Cost estimates for each insecticide

Total cost	α-cypermethrin	Deltamethrin	Pirimiphos-methyl
Estimated cost per village per spray	382	390	408
Projected annual cost per village	765	779	816
projected annual cost per square metre	0.06	0.41	0.19
The projected annual cost per sandfly reduced	0.37	0.97	0.58

## Discussion

Sandfly vector control remains a fundamental pillar in the elimination of visceral leishmaniasis and in achieving the WHO 2030 targets. This study aimed to evaluate the efficacy and cost-effectiveness of three insecticides, α-cypermethrin, pirimiphos-methyl, and deltamethrin, applied through ORS to reduce sandfly populations in leishmaniasis-endemic villages of Amudat District, Uganda. The results demonstrated substantial reductions across all intervention arms, with α-cypermethrin achieving the greatest decrease, followed by pirimiphos-methyl and deltamethrin. Cost-effectiveness analysis further showed that α-cypermethrin delivered the highest value for money, killing more sandflies per unit dollar than the other insecticides. These findings support the potential of ORS as a complementary vector control strategy in rural pastoral settings where exposure to sandflies is high [21].

The differences in performance among the insecticides can be explained by their distinct mechanisms of action. α-cypermethrin and deltamethrin are pyrethroids that disrupt voltage-gated sodium channels in insect nerve cells, providing sustained vector control over time. α-cypermethrin is known for its strong immediate knockdown effect, whereas deltamethrin typically shows longer residual activity[22]. Pirimiphos-methyl, an organophosphate, inhibits acetylcholinesterase, causing neuromuscular paralysis and death [23, 24]. These mechanistic differences may explain the faster and more pronounced reductions observed with α-cypermethrin and pirimiphos-methyl, whereas deltamethrin’s effectiveness may require longer exposure durations and indoor application to achieve optimal residual effect. Given the outdoor environment and unpredictability of sandfly contact with treated surfaces, agents with more rapid knockdown, like α-cypermethrin, may offer superior field performance under ORS conditions.

The superior performance of α-cypermethrin in this study aligns with previous research conducted in Brazil, where outdoor application similarly resulted in significant sandfly reductions [25]. Although our study used a single application, Barata et al. [25] reported that a double application produced even greater effects, suggesting that repeated rounds of α-cypermethrin may be valuable for sustained control. Laboratory findings also reinforce these results: Chaubey et al*. *[26] and Dinesh et al. [27] observed higher knockdown rates for α-cypermethrin compared to deltamethrin and pirimiphos-methyl. However, differences in application methods: single-round outdoor spraying versus multi-round indoor residual spraying (IRS), likely contribute to the variability in effect sizes across studies. Environmental factors, including dense vegetation and the presence of animal shelters, may have influenced contact rates between sandflies and sprayed surfaces, further modulating observed effectiveness [28].

Our findings support ORS as a potentially valuable intervention for sandfly control in pastoral and peridomestic environments, yet they also highlight important contrasts with IRS-based evidence. For example, our deltamethrin results (49% reduction over one month) differ from the 50–80% reductions reported in IRS studies [29]. This discrepancy is likely due to methodological and environmental factors: IRS involves indoor, stable surfaces with minimal weather interference, whereas ORS is affected by rainfall, sunlight, and vegetation cover, which degrade insecticide residues more rapidly, in addition to our study’s short, one-month follow-up period, as compared to the 15-month follow-up in the Chaves et al. study. Coleman et al. [30] similarly reported that harsh outdoor climatic conditions significantly reduce the residual activity of insecticides.

Our study further revealed a 60% reduction in the sandfly population after the application of pirimiphos-methyl in Katukwomok village, a finding consistent with the 62% sandfly density decline observed with alphacypermethrin, augmenting previous reports on the similar efficacy of these two insecticides. On the contrary, a laboratory study conducted in India found that sandflies were 98% susceptible to pirimiphos-methyl [27]. This noticeable difference denotes a contrast between controlled laboratory settings and real-world field conditions. Our field study was subject to various environmental factors that could not be controlled, unlike laboratory trials. In field trials where insecticides are applied on dense vegetation such as *Balanites aegyptiaca* and *Acacia seyal*,* which* may have limited the deposition of insecticide droplets on key resting surfaces, lower efficacies are not surprising [12, 15, 31–33]. Therefore, while ORS is feasible and effective, its performance is more environmentally dependent than IRS.

## Cost-effectiveness and policy implications

Cost-effectiveness assessment revealed that α-cypermethrin was the most economically favourable insecticide at $0.37 per sandfly reduced, compared with $0.58 for pirimiphos-methyl and $0.97 for deltamethrin. In low-resource regions like northeastern Uganda, where households already incur substantial direct and indirect costs from visceral leishmaniasis—estimated at $450 per episode [34]—selecting the most cost-efficient prevention tools is essential. Given its high reduction effect and affordability, α-cypermethrin represents a practical option for vector control departments seeking to scale up interventions. Integrating ORS with other strategies, such as insecticide-treated clothing or repellents for pastoral communities frequently exposed outdoors, could enhance protection and reduce disease risk.

## Limitations

The follow-up period was relatively short (one month), possibly underestimating long-term residual effects, particularly for deltamethrin. This study is limited by a relatively short 1-month follow-up period, which is insufficient to evaluate the residual efficacy of insecticides used in ORS. Future outdoor residual spraying interventions should therefore incorporate routine resistance monitoring as part of integrated vector management practices. 

## Conclusions

In summary, α-cypermethrin performed best in this pragmatic study in reducing sandfly populations and showed a higher cost-effectiveness value among the three insecticides tested. It therefore merits further study in a larger replicated trial. Seasonal weather patterns played a substantial role in shaping outcomes, underscoring the importance of aligning spraying activities with dry periods to maximise insecticide persistence. Based on these findings, α-cypermethrin emerges as a promising option for outdoor residual spraying in pastoral communities, where exposure to sandflies is primarily outdoors. However, given the limitations of this study, our results are exploratory, and could benefit from validation of larger multi-cluster trials to yield definitive recommendations.

## Supplementary Information

Additional file1.

## Data Availability

Raw data were generated in the Amudat District Local government. Derived data supporting the findings of this study are available from the corresponding author, KL, on request.

## Abbreviations

VL: Visceral leishmaniasis
ORS: Outdoor residual spraying
CDC: Center for Disease Control and Prevention
IRR: Incidence rate ratio
WHO: World Health Organization
CRCT: Cluster randomised controlled trials
SNOSE: Sequentially numbered opaque sealed envelopes
a.i: Active ingredient
PPE: Personal protective equipment
DID: Difference in difference
USD: United States dollar
IRS: Indoor residual spraying
