# Supplementary figures and images for: Efficacy and cost-effectiveness of outdoor residual spraying with α-cypermethrin, pirimiphos-methyl, and deltamethrin against sand flies: a pragmatic cluster randomised controlled field trial in Uganda
Source: Trop Med Health. 2026 May 27;54:100. doi: 10.1186/s41182-026-00985-9 (PMC13217796; doi:10.1186/s41182-026-00985-9)

Supplemental file for Autocorrelations


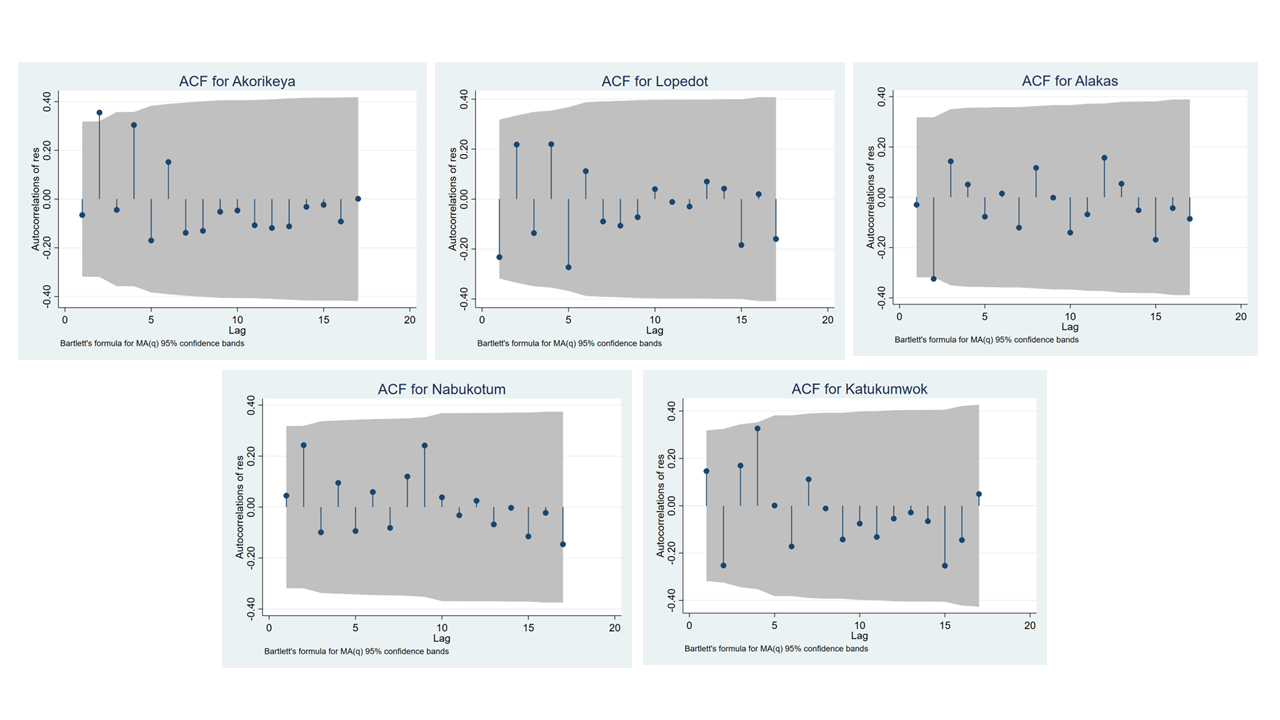

Supplement: Supplementary file 1 — Additional file1. [file 41182_2026_985_MOESM1_ESM.docx]
